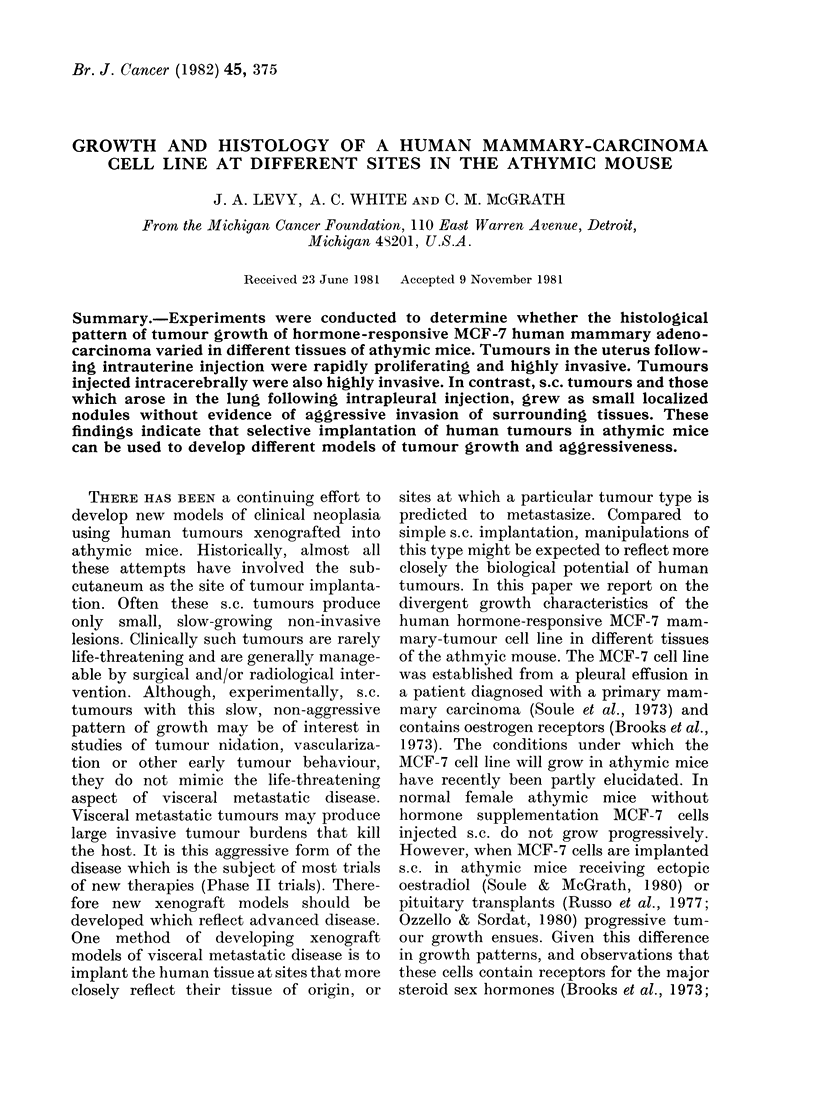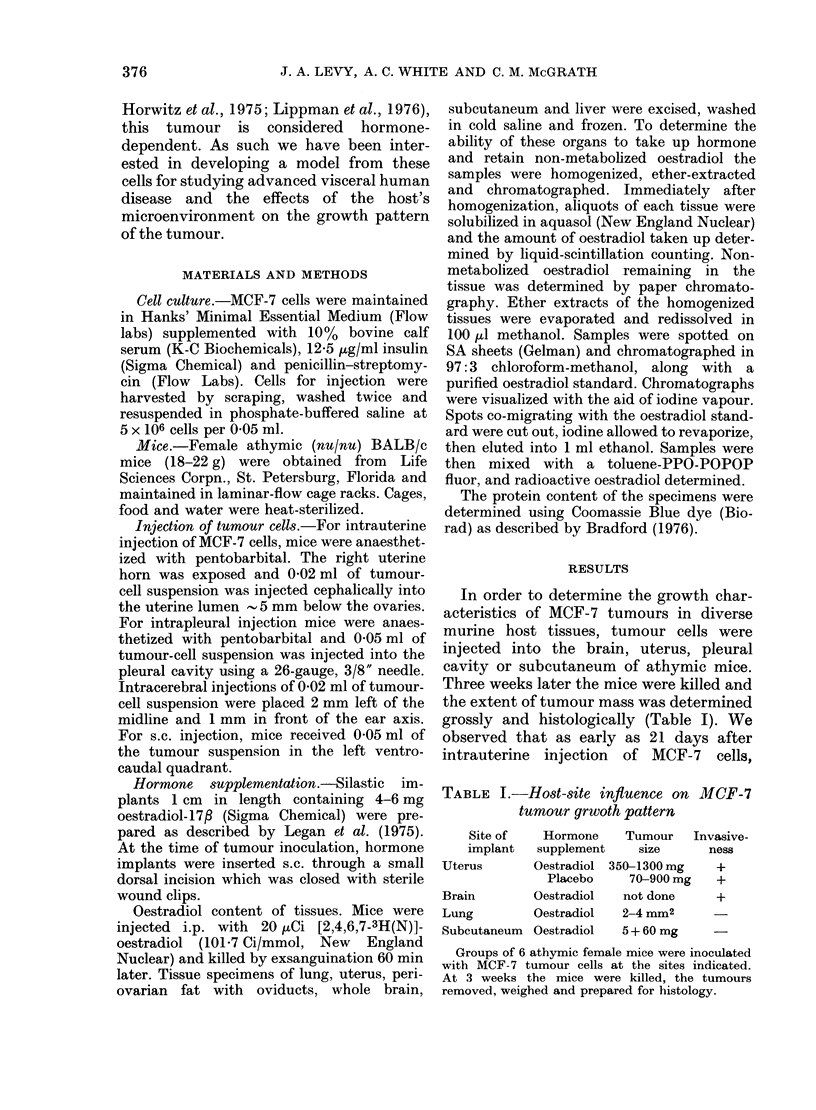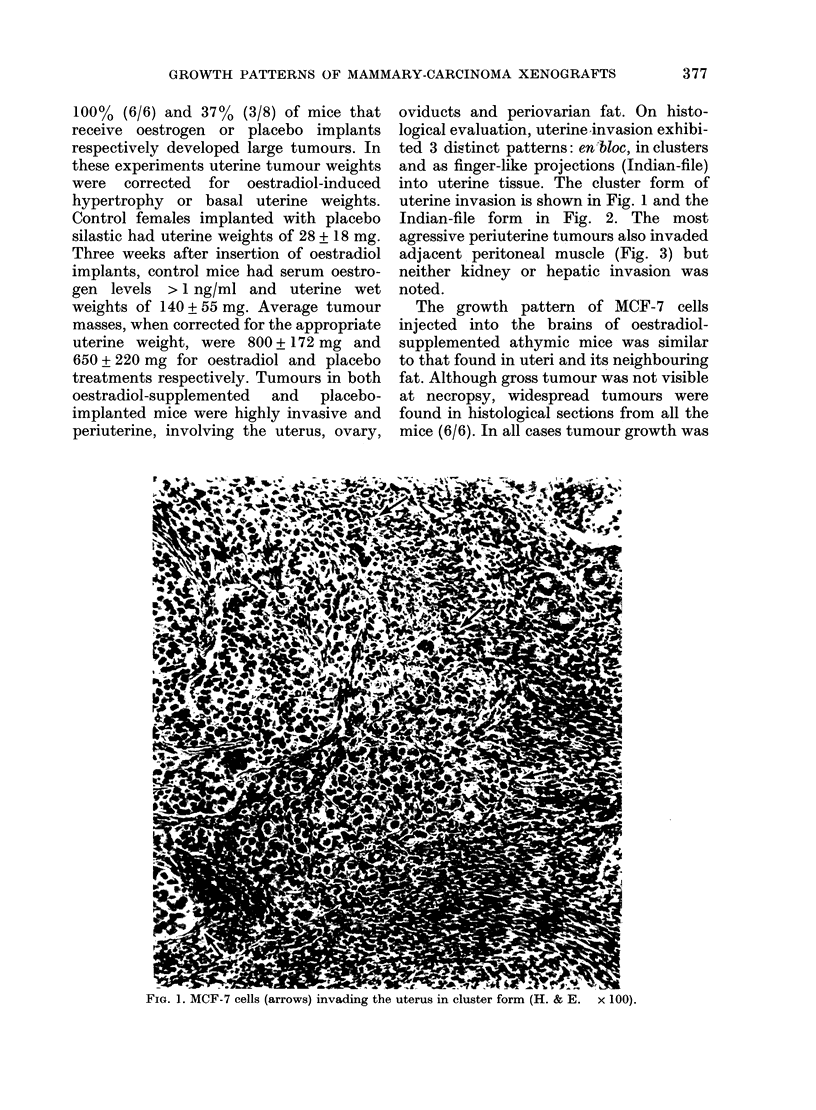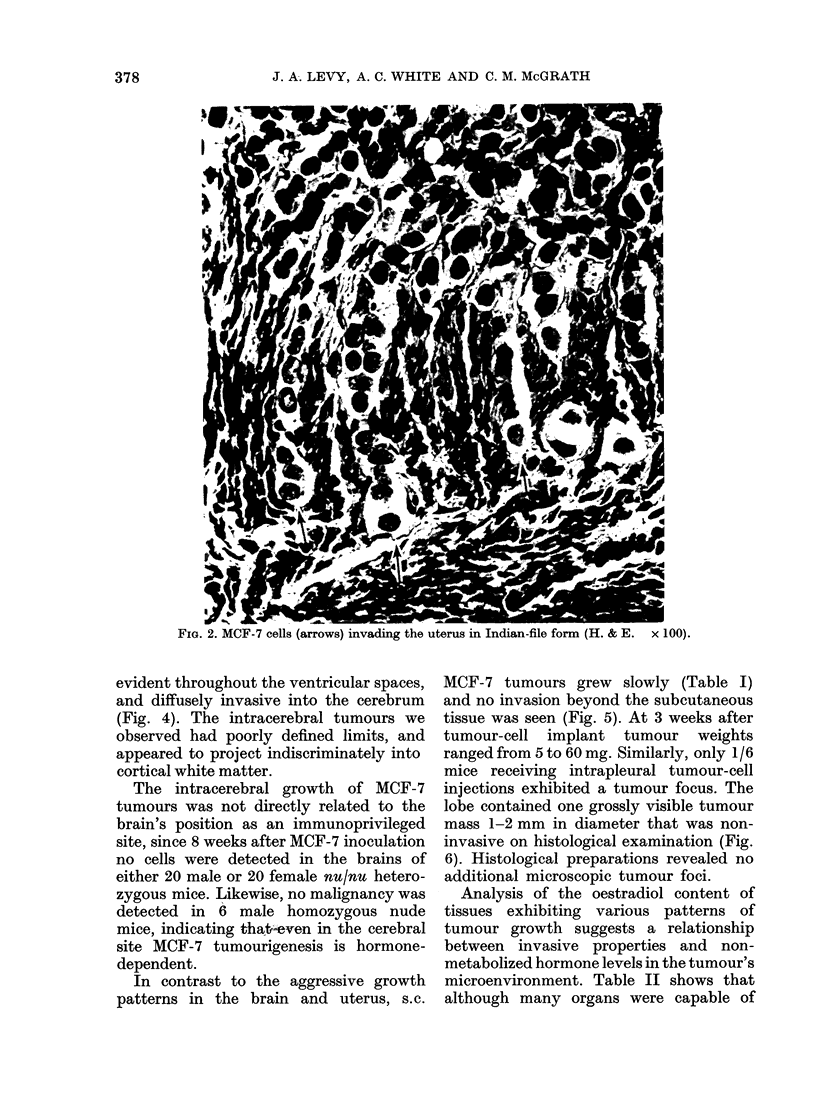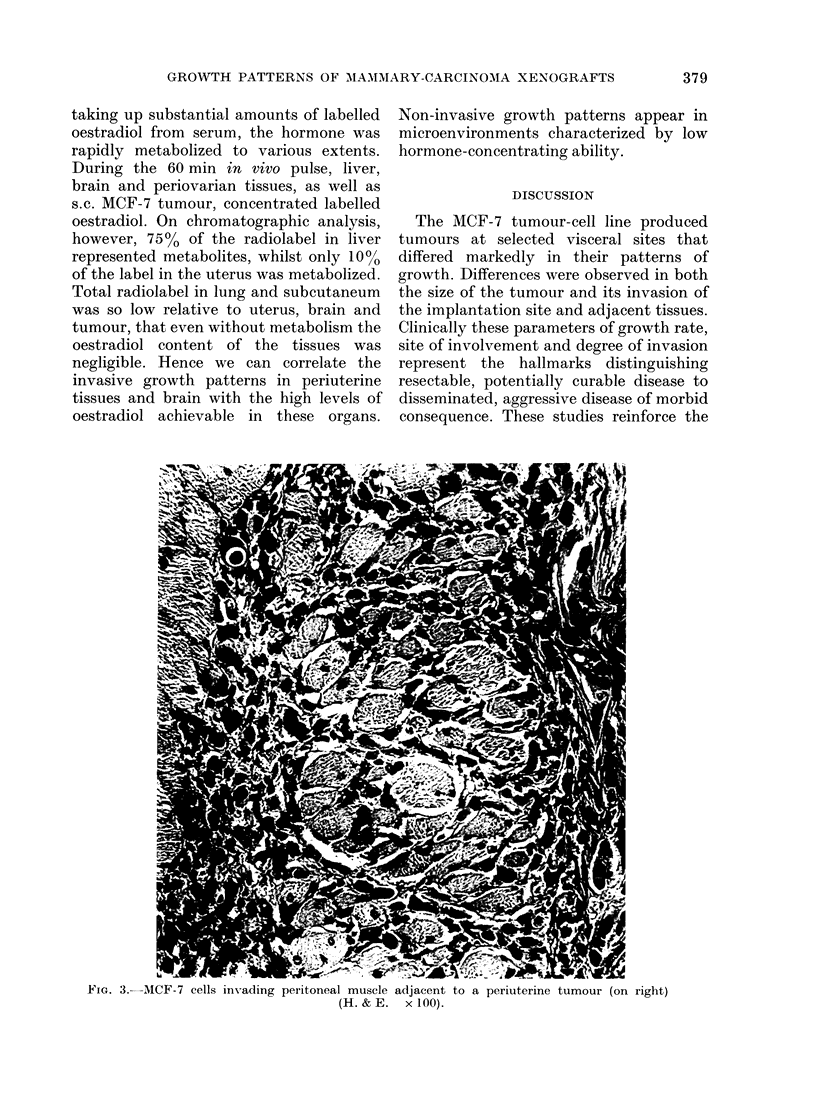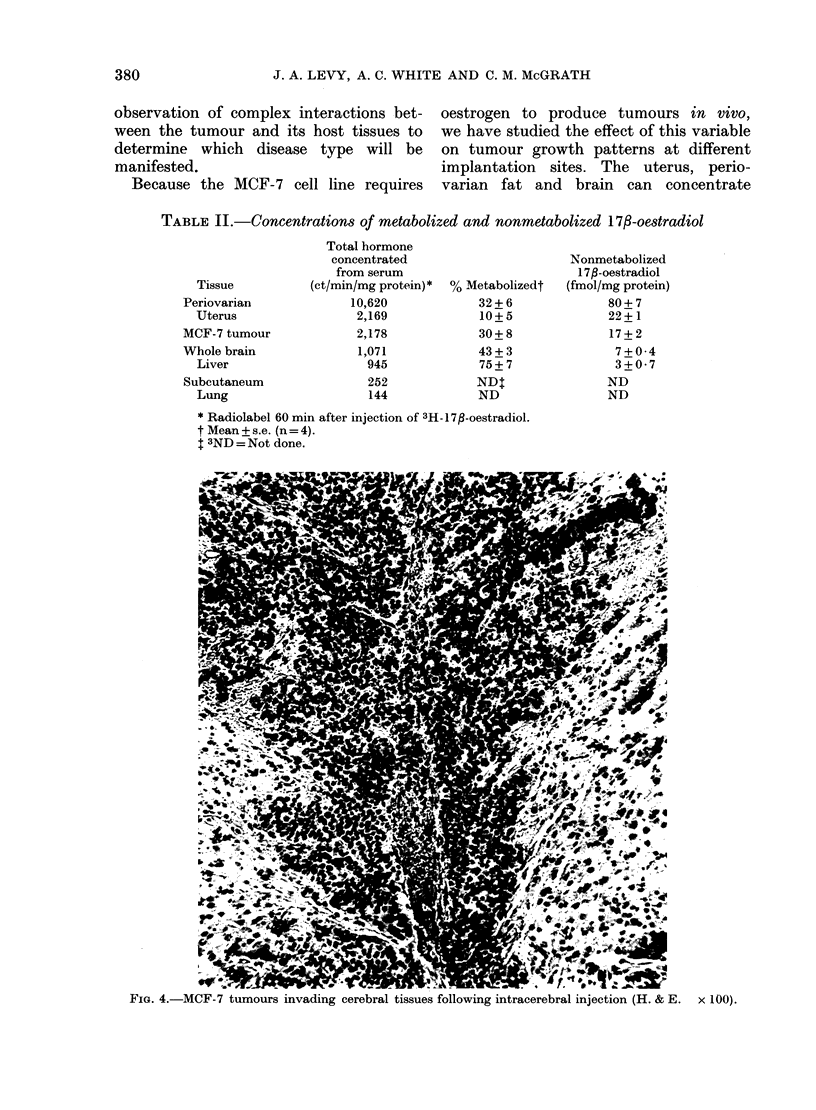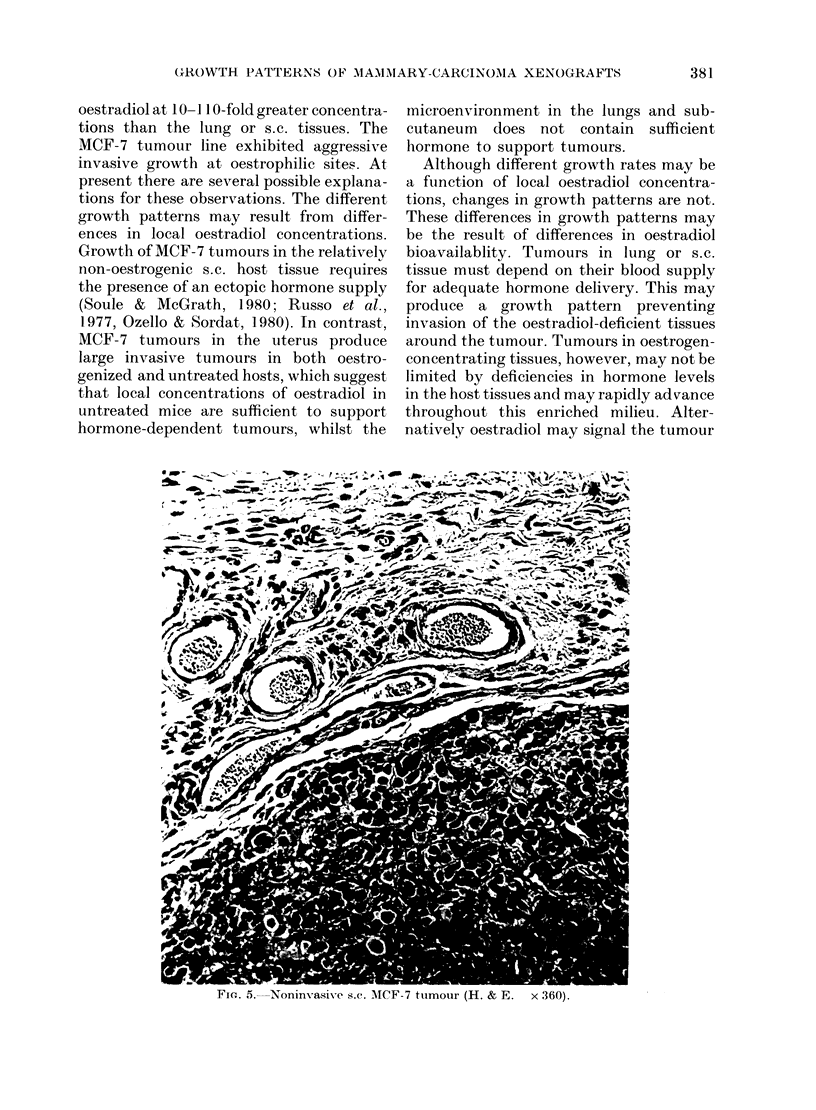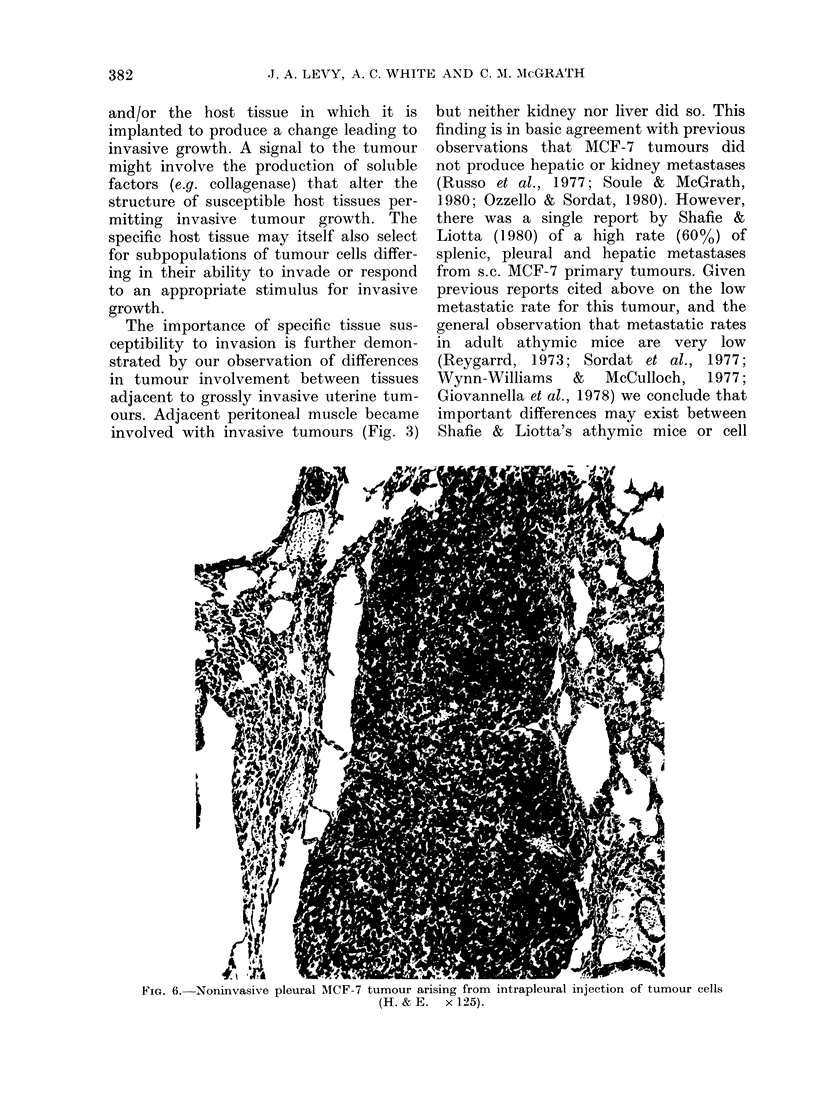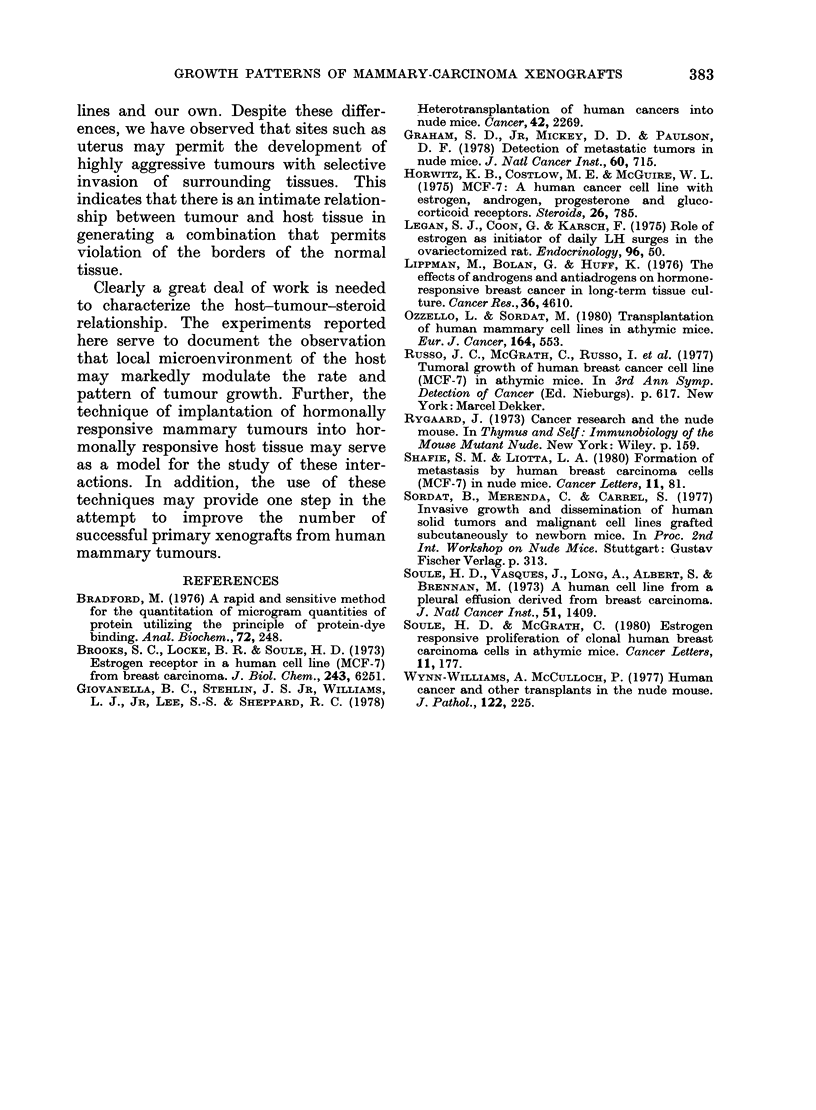# Growth and histology of a human mammary-carcinoma cell line at different sites in the athymic mouse.

**DOI:** 10.1038/bjc.1982.64

**Published:** 1982-03

**Authors:** J. A. Levy, A. C. White, C. M. McGrath

## Abstract

**Images:**


*Br. J. Cancer* (1982) **45**, 375

### GROWTH AND HISTOLOGY OF A HUMAN MAMMARY-CARCINOMA CELL LINE AT DIFFERENT SITES IN THE ATHYMIC MOUSE

J. A. LEVY, A. C. WHITE AND C. M. McGRATH

*From the Michigan Cancer Foundation,* 110 *East Warren Avenue, Detroit, Michigan* 48201, *U.S.A.*

Received 23 June 1981    Accepted 9 November 1981

**Summary.—Experiments were conducted to determine whether the histological pattern of tumour growth of hormone-responsive MCF-7 human mammary adenocarcinoma varied in different tissues of athymic mice. Tumours in the uterus following intrauterine injection were rapidly proliferating and highly invasive. Tumours injected intracerebrally were also highly invasive. In contrast, s.c. tumours and those which arose in the lung following intrapleural injection, grew as small localized nodules without evidence of aggressive invasion of surrounding tissues. These findings indicate that selective implantation of human tumours in athymic mice can be used to develop different models of tumour growth and aggressiveness.**

THERE HAS BEEN a continuing effort to develop new models of clinical neoplasia using human tumours xenografted into athymic mice. Historically, almost all these attempts have involved the subcutaneum as the site of tumour implantation. Often these s.c. tumours produce only small, slow-growing non-invasive lesions. Clinically such tumours are rarely life-threatening and are generally manageable by surgical and/or radiological intervention. Although, experimentally, s.c. tumours with this slow, non-aggressive pattern of growth may be of interest in studies of tumour nidation, vascularization or other early tumour behaviour, they do not mimic the life-threatening aspect of visceral metastatic disease. Visceral metastatic tumours may produce large invasive tumour burdens that kill the host. It is this aggressive form of the disease which is the subject of most trials of new therapies (Phase II trials). Therefore new xenograft models should be developed which reflect advanced disease. One method of developing xenograft models of visceral metastatic disease is to implant the human tissue at sites that more closely reflect their tissue of origin, or sites at which a particular tumour type is predicted to metastasize. Compared to simple s.c. implantation, manipulations of this type might be expected to reflect more closely the biological potential of human tumours. In this paper we report on the divergent growth characteristics of the human hormone-responsive MCF-7 mammary-tumour cell line in different tissues of the athmyic mouse. The MCF-7 cell line was established from a pleural effusion in a patient diagnosed with a primary mammary carcinoma (Soule *et al.*, 1973) and contains oestrogen receptors (Brooks *et al.*, 1973). The conditions under which the MCF-7 cell line will grow in athymic mice have recently been partly elucidated. In normal female athymic mice without hormone supplementation MCF-7 cells injected s.c. do not grow progressively. However, when MCF-7 cells are implanted s.c. in athymic mice receiving ectopic oestradiol (Soule & McGrath, 1980) or pituitary transplants (Russo *et al.*, 1977; Ozzello & Sordat, 1980) progressive tumour growth ensues. Given this difference in growth patterns, and observations that these cells contain receptors for the major steroid sex hormones (Brooks *et al.*, 1973;

Horwitz *et al.*, 1975; Lippman *et al.*, 1976), this tumour is considered hormone-dependent. As such we have been interested in developing a model from these cells for studying advanced visceral human disease and the effects of the host's microenvironment on the growth pattern of the tumour.

### MATERIALS AND METHODS

*Cell culture.*—MCF-7 cells were maintained in Hanks' Minimal Essential Medium (Flow labs) supplemented with 10% bovine calf serum (K-C Biochemicals), 12·5 $\mu$g/ml insulin (Sigma Chemical) and penicillin–streptomycin (Flow Labs). Cells for injection were harvested by scraping, washed twice and resuspended in phosphate-buffered saline at $5 \times 10^6$ cells per 0·05 ml.

*Mice.*—Female athymic (*nu/nu*) BALB/c mice (18–22 g) were obtained from Life Sciences Corpn., St. Petersburg, Florida and maintained in laminar-flow cage racks. Cages, food and water were heat-sterilized.

*Injection of tumour cells.*—For intrauterine injection of MCF-7 cells, mice were anaesthetized with pentobarbital. The right uterine horn was exposed and 0·02 ml of tumour-cell suspension was injected cephalically into the uterine lumen ~5 mm below the ovaries. For intrapleural injection mice were anaesthetized with pentobarbital and 0·05 ml of tumour-cell suspension was injected into the pleural cavity using a 26-gauge, 3/8″ needle. Intracerebral injections of 0·02 ml of tumour-cell suspension were placed 2 mm left of the midline and 1 mm in front of the ear axis. For s.c. injection, mice received 0·05 ml of the tumour suspension in the left ventro-caudal quadrant.

*Hormone supplementation.*—Silastic implants 1 cm in length containing 4–6 mg oestradiol-17$\beta$ (Sigma Chemical) were prepared as described by Legan *et al.* (1975). At the time of tumour inoculation, hormone implants were inserted s.c. through a small dorsal incision which was closed with sterile wound clips.

Oestradiol content of tissues. Mice were injected i.p. with 20 $\mu$Ci [2,4,6,7-$^3$H(N)]-oestradiol (101·7 Ci/mmol, New England Nuclear) and killed by exsanguination 60 min later. Tissue specimens of lung, uterus, periovarian fat with oviducts, whole brain, subcutaneum and liver were excised, washed in cold saline and frozen. To determine the ability of these organs to take up hormone and retain non-metabolized oestradiol the samples were homogenized, ether-extracted and chromatographed. Immediately after homogenization, aliquots of each tissue were solubilized in aquasol (New England Nuclear) and the amount of oestradiol taken up determined by liquid-scintillation counting. Non-metabolized oestradiol remaining in the tissue was determined by paper chromatography. Ether extracts of the homogenized tissues were evaporated and redissolved in 100 $\mu$l methanol. Samples were spotted on SA sheets (Gelman) and chromatographed in 97:3 chloroform-methanol, along with a purified oestradiol standard. Chromatographs were visualized with the aid of iodine vapour. Spots co-migrating with the oestradiol standard were cut out, iodine allowed to revaporize, then eluted into 1 ml ethanol. Samples were then mixed with a toluene-PPO-POPOP fluor, and radioactive oestradiol determined.

The protein content of the specimens were determined using Coomassie Blue dye (Bio-rad) as described by Bradford (1976).

### RESULTS

In order to determine the growth characteristics of MCF-7 tumours in diverse murine host tissues, tumour cells were injected into the brain, uterus, pleural cavity or subcutaneum of athymic mice. Three weeks later the mice were killed and the extent of tumour mass was determined grossly and histologically (Table I). We observed that as early as 21 days after intrauterine injection of MCF-7 cells,

TABLE I.—*Host-site influence on MCF-7 tumour grwoth pattern*

| Site of implant | Hormone supplement | Tumour size | Invasiveness |
|---|---|---|---|
| Uterus | Oestradiol | 350–1300 mg | + |
| | Placebo | 70–900 mg | + |
| Brain | Oestradiol | not done | + |
| Lung | Oestradiol | 2–4 mm$^2$ | — |
| Subcutaneum | Oestradiol | 5 + 60 mg | — |

Groups of 6 athymic female mice were inoculated with MCF-7 tumour cells at the sites indicated. At 3 weeks the mice were killed, the tumours removed, weighed and prepared for histology.

100% (6/6) and 37% (3/8) of mice that receive oestrogen or placebo implants respectively developed large tumours. In these experiments uterine tumour weights were corrected for oestradiol-induced hypertrophy or basal uterine weights. Control females implanted with placebo silastic had uterine weights of 28 ± 18 mg. Three weeks after insertion of oestradiol implants, control mice had serum oestrogen levels > 1 ng/ml and uterine wet weights of 140 ± 55 mg. Average tumour masses, when corrected for the appropriate uterine weight, were 800 ± 172 mg and 650 ± 220 mg for oestradiol and placebo treatments respectively. Tumours in both oestradiol-supplemented and placebo-implanted mice were highly invasive and periuterine, involving the uterus, ovary, oviducts and periovarian fat. On histological evaluation, uterine invasion exhibited 3 distinct patterns: *en bloc*, in clusters and as finger-like projections (Indian-file) into uterine tissue. The cluster form of uterine invasion is shown in Fig. 1 and the Indian-file form in Fig. 2. The most agressive periuterine tumours also invaded adjacent peritoneal muscle (Fig. 3) but neither kidney or hepatic invasion was noted.

The growth pattern of MCF-7 cells injected into the brains of oestradiol-supplemented athymic mice was similar to that found in uteri and its neighbouring fat. Although gross tumour was not visible at necropsy, widespread tumours were found in histological sections from all the mice (6/6). In all cases tumour growth was

FIG. 1. MCF-7 cells (arrows) invading the uterus in cluster form (H. & E. × 100).

FIG. 2. MCF-7 cells (arrows) invading the uterus in Indian-file form (H. & E. ×100).

evident throughout the ventricular spaces, and diffusely invasive into the cerebrum (Fig. 4). The intracerebral tumours we observed had poorly defined limits, and appeared to project indiscriminately into cortical white matter.

The intracerebral growth of MCF-7 tumours was not directly related to the brain's position as an immunoprivileged site, since 8 weeks after MCF-7 inoculation no cells were detected in the brains of either 20 male or 20 female *nu/nu* heterozygous mice. Likewise, no malignancy was detected in 6 male homozygous nude mice, indicating that even in the cerebral site MCF-7 tumourigenesis is hormone-dependent.

In contrast to the aggressive growth patterns in the brain and uterus, s.c. MCF-7 tumours grew slowly (Table I) and no invasion beyond the subcutaneous tissue was seen (Fig. 5). At 3 weeks after tumour-cell implant tumour weights ranged from 5 to 60 mg. Similarly, only 1/6 mice receiving intrapleural tumour-cell injections exhibited a tumour focus. The lobe contained one grossly visible tumour mass 1–2 mm in diameter that was non-invasive on histological examination (Fig. 6). Histological preparations revealed no additional microscopic tumour foci.

Analysis of the oestradiol content of tissues exhibiting various patterns of tumour growth suggests a relationship between invasive properties and non-metabolized hormone levels in the tumour's microenvironment. Table II shows that although many organs were capable of taking up substantial amounts of labelled oestradiol from serum, the hormone was rapidly metabolized to various extents. During the 60 min *in vivo* pulse, liver, brain and periovarian tissues, as well as s.c. MCF-7 tumour, concentrated labelled oestradiol. On chromatographic analysis, however, 75% of the radiolabel in liver represented metabolites, whilst only 10% of the label in the uterus was metabolized. Total radiolabel in lung and subcutaneum was so low relative to uterus, brain and tumour, that even without metabolism the oestradiol content of the tissues was negligible. Hence we can correlate the invasive growth patterns in periuterine tissues and brain with the high levels of oestradiol achievable in these organs. Non-invasive growth patterns appear in microenvironments characterized by low hormone-concentrating ability.

### DISCUSSION

The MCF-7 tumour-cell line produced tumours at selected visceral sites that differed markedly in their patterns of growth. Differences were observed in both the size of the tumour and its invasion of the implantation site and adjacent tissues. Clinically these parameters of growth rate, site of involvement and degree of invasion represent the hallmarks distinguishing resectable, potentially curable disease to disseminated, aggressive disease of morbid consequence. These studies reinforce the

FIG. 3.—MCF-7 cells invading peritoneal muscle adjacent to a periuterine tumour (on right) (H. & E. ×100).

observation of complex interactions between the tumour and its host tissues to determine which disease type will be manifested.

Because the MCF-7 cell line requires oestrogen to produce tumours *in vivo*, we have studied the effect of this variable on tumour growth patterns at different implantation sites. The uterus, periovarian fat and brain can concentrate

TABLE II.—*Concentrations of metabolized and nonmetabolized 17β-oestradiol*

| Tissue | Total hormone concentrated from serum (ct/min/mg protein)* | % Metabolized† | Nonmetabolized 17β-oestradiol (fmol/mg protein) |
|---|---|---|---|
| Periovarian | 10,620 | 32 ± 6 | 80 ± 7 |
| Uterus | 2,169 | 10 ± 5 | 22 ± 1 |
| MCF-7 tumour | 2,178 | 30 ± 8 | 17 ± 2 |
| Whole brain | 1,071 | 43 ± 3 | 7 ± 0·4 |
| Liver | 945 | 75 ± 7 | 3 ± 0·7 |
| Subcutaneum | 252 | ND‡ | ND |
| Lung | 144 | ND | ND |

* Radiolabel 60 min after injection of $^{3}$H-17β-oestradiol.

† Mean ± s.e. (n = 4).

‡ $^{3}$ND = Not done.

FIG. 4.—MCF-7 tumours invading cerebral tissues following intracerebral injection (H. & E. × 100).

oestradiol at 10–110-fold greater concentrations than the lung or s.c. tissues. The MCF-7 tumour line exhibited aggressive invasive growth at oestrophilic sites. At present there are several possible explanations for these observations. The different growth patterns may result from differences in local oestradiol concentrations. Growth of MCF-7 tumours in the relatively non-oestrogenic s.c. host tissue requires the presence of an ectopic hormone supply (Soule & McGrath, 1980; Russo *et al.*, 1977, Ozello & Sordat, 1980). In contrast, MCF-7 tumours in the uterus produce large invasive tumours in both oestrogenized and untreated hosts, which suggest that local concentrations of oestradiol in untreated mice are sufficient to support hormone-dependent tumours, whilst the microenvironment in the lungs and subcutaneum does not contain sufficient hormone to support tumours.

Although different growth rates may be a function of local oestradiol concentrations, changes in growth patterns are not. These differences in growth patterns may be the result of differences in oestradiol bioavailablity. Tumours in lung or s.c. tissue must depend on their blood supply for adequate hormone delivery. This may produce a growth pattern preventing invasion of the oestradiol-deficient tissues around the tumour. Tumours in oestrogen-concentrating tissues, however, may not be limited by deficiencies in hormone levels in the host tissues and may rapidly advance throughout this enriched milieu. Alternatively oestradiol may signal the tumour

FIG. 5.—Noninvasive s.c. MCF-7 tumour (H. & E. ×360).

and/or the host tissue in which it is implanted to produce a change leading to invasive growth. A signal to the tumour might involve the production of soluble factors (*e.g.* collagenase) that alter the structure of susceptible host tissues permitting invasive tumour growth. The specific host tissue may itself also select for subpopulations of tumour cells differing in their ability to invade or respond to an appropriate stimulus for invasive growth.

The importance of specific tissue susceptibility to invasion is further demonstrated by our observation of differences in tumour involvement between tissues adjacent to grossly invasive uterine tumours. Adjacent peritoneal muscle became involved with invasive tumours (Fig. 3) but neither kidney nor liver did so. This finding is in basic agreement with previous observations that MCF-7 tumours did not produce hepatic or kidney metastases (Russo *et al.*, 1977; Soule & McGrath, 1980; Ozzello & Sordat, 1980). However, there was a single report by Shafie & Liotta (1980) of a high rate (60%) of splenic, pleural and hepatic metastases from s.c. MCF-7 primary tumours. Given previous reports cited above on the low metastatic rate for this tumour, and the general observation that metastatic rates in adult athymic mice are very low (Reygarrd, 1973; Sordat *et al.*, 1977; Wynn-Williams & McCulloch, 1977; Giovannella *et al.*, 1978) we conclude that important differences may exist between Shafie & Liotta's athymic mice or cell

FIG. 6.—Noninvasive pleural MCF-7 tumour arising from intrapleural injection of tumour cells (H. & E. ×125).

lines and our own. Despite these differences, we have observed that sites such as uterus may permit the development of highly aggressive tumours with selective invasion of surrounding tissues. This indicates that there is an intimate relationship between tumour and host tissue in generating a combination that permits violation of the borders of the normal tissue.

Clearly a great deal of work is needed to characterize the host–tumour–steroid relationship. The experiments reported here serve to document the observation that local microenvironment of the host may markedly modulate the rate and pattern of tumour growth. Further, the technique of implantation of hormonally responsive mammary tumours into hormonally responsive host tissue may serve as a model for the study of these interactions. In addition, the use of these techniques may provide one step in the attempt to improve the number of successful primary xenografts from human mammary tumours.

### REFERENCES

BRADFORD, M. (1976) A rapid and sensitive method for the quantitation of microgram quantities of protein utilizing the principle of protein-dye binding. *Anal. Biochem.*, **72**, 248.

BROOKS, S. C., LOCKE, B. R. & SOULE, H. D. (1973) Estrogen receptor in a human cell line (MCF-7) from breast carcinoma. *J. Biol. Chem.*, **243**, 6251.

GIOVANELLA, B. C., STEHLIN, J. S. JR, WILLIAMS, L. J., JR, LEE, S.-S. & SHEPPARD, R. C. (1978) Heterotransplantation of human cancers into nude mice. *Cancer*, **42**, 2269.

GRAHAM, S. D., JR, MICKEY, D. D. & PAULSON, D. F. (1978) Detection of metastatic tumors in nude mice. *J. Natl Cancer Inst.*, **60**, 715.

HORWITZ, K. B., COSTLOW, M. E. & MCGUIRE, W. L. (1975) MCF-7: A human cancer cell line with estrogen, androgen, progesterone and glucocorticoid receptors. *Steroids*, **26**, 785.

LEGAN, S. J., COON, G. & KARSCH, F. (1975) Role of estrogen as initiator of daily LH surges in the ovariectomized rat. *Endocrinology*, **96**, 50.

LIPPMAN, M., BOLAN, G. & HUFF, K. (1976) The effects of androgens and antiadrogens on hormone-responsive breast cancer in long-term tissue culture. *Cancer Res.*, **36**, 4610.

OZZELLO, L. & SORDAT, M. (1980) Transplantation of human mammary cell lines in athymic mice. *Eur. J. Cancer*, **164**, 553.

RUSSO, J. C., MCGRATH, C., RUSSO, I. *et al.* (1977) Tumoral growth of human breast cancer cell line (MCF-7) in athymic mice. In *3rd Ann Symp. Detection of Cancer* (Ed. Nieburgs). p. 617. New York: Marcel Dekker.

RYGAARD, J. (1973) Cancer research and the nude mouse. In *Thymus and Self: Immunobiology of the Mouse Mutant Nude.* New York: Wiley. p. 159.

SHAFIE, S. M. & LIOTTA, L. A. (1980) Formation of metastasis by human breast carcinoma cells (MCF-7) in nude mice. *Cancer Letters*, **11**, 81.

SORDAT, B., MERENDA, C. & CARREL, S. (1977) Invasive growth and dissemination of human solid tumors and malignant cell lines grafted subcutaneously to newborn mice. In *Proc. 2nd Int. Workshop on Nude Mice.* Stuttgart: Gustav Fischer Verlag. p. 313.

SOULE, H. D., VASQUES, J., LONG, A., ALBERT, S. & BRENNAN, M. (1973) A human cell line from a pleural effusion derived from breast carcinoma. *J. Natl Cancer Inst.*, **51**, 1409.

SOULE, H. D. & MCGRATH, C. (1980) Estrogen responsive proliferation of clonal human breast carcinoma cells in athymic mice. *Cancer Letters*, **11**, 177.

WYNN-WILLIAMS, A. MCCULLOCH, P. (1977) Human cancer and other transplants in the nude mouse. *J. Pathol.*, **122**, 225.